# mDAG: a web-based tool for analyzing microarray data with multiple treatments

**DOI:** 10.1186/1471-2105-12-S7-A7

**Published:** 2011-08-05

**Authors:** Vinhthuy T Phan, Nam S Vo, Thomas R Sutter

**Affiliations:** 1 Department of Computer Science, University of Memphis, Memphis, TN, 38152, USA; 2 Bioinformatics Program, University of Memphis, Memphis, TN, 38152, USA; 3 Department of Biological Sciences, University of Memphis, Memphis, TN, 38152, USA

## Background

In microarray experiments involving multiple treatments, pairwise comparisons between all pairs of treatments are desirable but expensive. To cope with this, we previously introduced a method that performed all pairwise comparisons in a *post hoc* manner. This method employs directed graphs to represent gene response to pairs of treatments. It has been applied and found useful in identifying and differentiating genes sharing similar functional pathways [1,2].

## Results

mDAG is a web-based software based on this method. mDAG allows users to upload microarray data in GCT format through a web interface. From this data, the application performs calculations to assign graphical patterns to genes and outputs images and textual data for further analyses. These graphical patterns carry specific meanings in terms of how genes respond to pairs of treatments. The application is implemented using Python and web2py.

mDAG is available at http://cetus.cs.memphis.edu:8080/mDAG.

## Conclusion

For experiments involved multiple treatments and replicates, mDAG allows researchers to analyze and visualize in graphical representations relationships of gene interactions to all pairs of treatments. The software can be used online or off-line.
